# Burden and factors influencing intestinal parasitic infections among food handlers in Gondar City, Northwest Ethiopia

**DOI:** 10.3389/fpubh.2024.1362086

**Published:** 2024-06-11

**Authors:** Michael Getie, Gizeaddis Belay, Azanaw Amare, Wondwossen Abebe, Teshiwal Deress

**Affiliations:** ^1^Department of Medical Microbiology, Amhara National Regional State Public Health Institute, Bahir Dar, Ethiopia; ^2^Department of Medical Microbiology, School of Biomedical and Laboratory Sciences, College of Medicine and Health Sciences, University of Gondar, Gondar, Ethiopia; ^3^Department of Quality Assurance and Laboratory Management, School of Biomedical and Laboratory Sciences, College of Medicine and Health Sciences, University of Gondar, Gondar, Ethiopia

**Keywords:** food handlers, intestinal parasites, food sanitation, Gondar, Ethiopia

## Abstract

**Background:**

Intestinal parasitic infections pose significant global health challenges, particularly in developing countries. Asymptomatic infections often present a considerable burden with food handlers serving as potential carriers. In Ethiopia, the prevalence of these parasites varies across regions, and accurate data in the study area is lacking. Therefore, this study aimed to investigate the prevalence of intestinal parasites among food handlers working in hotels and restaurants in Gondar City, Northwest Ethiopia.

**Methods:**

A cross-sectional study collected stool samples from food handlers alongside a structured questionnaire gathering socio-demographic and hygiene practice information. Stool specimens were screened for intestinal parasites using direct wet mount and formol-ether concentration techniques. The collected data were checked for completeness, entered into EpiData software version 3.1, and exported to SPSS version 20 for analysis. A multivariable logistic regression analysis was deemed statistically significant if the *p*-value was less than 0.05.

**Results:**

A total of 257 food handlers working in hotels and restaurants in Gondar City participated in the study. Of these, 33.5% (86/257) were found positive for one or more intestinal parasites, with a 95% confidence interval (CI) of 28.0–39.5%. The study identified nine types of intestinal parasites, with *E. histolytica/dispar* (8.2%, 21/257) and Ascaris lumbricoides (6.6%, 17/257) being the predominant parasites, followed by hookworm (3.5%, 9/257) and *S. mansoni* (2.3%, 6/257). The prevalence of mixed infections was 9.3% (24/257). A significant association was observed between intestinal parasitic infection and the educational level of food handlers.

**Conclusion:**

In this study, a high prevalence of intestinal parasites was detected indicating poor hygiene practices of the food handlers at the study site. Even the prevalence of mixed infections was high. Regular training, strict adherence to personal hygiene and food-handling practices, and routine inspections and medical checkups for food handlers are crucial.

## Background

Gastrointestinal parasitic infections are globally distributed and have a significant impact on developing nations (1). Certain parasites possess the ability to afflict both humans and animals, posing a severe threat to the well-being of both species (2). Factors such as inadequate personal hygiene, suboptimal environmental sanitation, and various socio-economic, demographic, and health-related behaviors contribute to the transmission of these infections (3, 4). The health status and hygiene practices of food handlers play a vital role in determining the contamination of food and beverages, particularly in regions with weak regulatory frameworks for food hygiene (5–8). Food handlers can act as carriers and disseminators of enteropathogens, directly or indirectly contaminating food and posing a potential threat to consumer health (7, 9, 10). The area beneath fingernails, which is challenging to clean, harbors a high concentration of microorganisms, further increasing the risk of contamination (6, 11–14). Infected food handlers with poor personal hygiene can serve as significant sources of transmission to society. These handlers are often asymptomatic carriers who are unaware of their role in spreading infections, which hampers effective control and elimination efforts (15). Intestinal parasitic infections (IPIs) often do not exhibit clinical signs and symptoms, and they have several potential carriers, such as food handlers, making eradication and control challenging (16).

Poor hand hygiene and the absence of food safety training are significant factors contributing to the high prevalence of IPIs among food handlers (17). Regular medical check-ups and handwashing practices are protective measures that significantly reduce the risk of infection (13, 17). Foodborne intestinal parasitic diseases are the major causes of morbidity and high death rates globally (18). Intestinal parasites impose a substantial global burden, affecting approximately 3.5 billion people annually and causing over 200,000 reported deaths worldwide (19). Developing countries, especially those in Sub-Saharan Africa, bear a higher burden of intestinal parasites compared to developed nations (20). In Ethiopia, approximately 50,000 deaths per year are attributed to intestinal parasites (21). These infections not only cause morbidity and mortality but also have long-term effects on the health, nutritional status, and overall development of affected individuals (22). Gastrointestinal illnesses impose a significant social and economic burden, particularly in low and middle-income countries, where they are among the leading causes of morbidity and mortality (23). The loss of productivity due to illness affects not only individuals but also businesses and economies at large. The financial impact is further exacerbated by the high prevalence of such diseases in developing nations, where many people still consume contaminated water and lack proper sanitation (24). Food handlers play a critical role in the transmission of intestinal parasites, especially when they do not practice proper hand hygiene after using the toilet or before food preparation (13, 25).

In developing countries like Ethiopia, urbanization has led to increased patronage of food service establishments, where the health and hygiene practices of food handlers are critical in preventing food contamination. Unfortunately, food handlers are often employed without screening for infections that can be transmitted due to poor hygiene, such as intestinal parasites (6). Studies conducted in different settings have reported a high prevalence of intestinal parasites among food handlers, ranging from 29 to 63% (11, 13, 26). Studies in different parts of Ethiopia have revealed a wide variability in the prevalence of intestinal parasites among food handlers (27, 28), with rates ranging from 14.5 to 46.3% (6, 17, 19, 21, 29–33). A recent review in Ethiopia found that 33.6% of food handlers in food establishments were infected with *E. histolytica/dispar* and Ascaris lumbricoides (13). The diversity of factors contributing to the spread of intestinal parasites, such as water sources, personal hygiene practices, and environmental sanitation, underscores the complexity of controlling these infections (25). Moreover, the diverse nature of these parasite species continues to influence the strategies employed to reduce and combat the infection (34).

One of the main challenges in implementing effective public health measures to combat IPIs is the lack of comprehensive and up-to-date data. Many studies, including those conducted in Gondar City, have limitations in scope and may not be generalizable to the broader population (25, 35). This lack of data hinders the ability of local health planners to develop and implement appropriate intervention measures tailored to the specific risk factors identified in different populations. Furthermore, given the widespread prevalence of IPIs with regional variations, there is a pressing need for periodic assessments to guide future interventions, particularly among high-risk groups such as food handlers. Therefore, this study aimed to evaluate the prevalence of IPIs and the factors associated with food handlers working in hotels and restaurants in Gondar City, Northwest Ethiopia.

## Rationale

The growing popularity of Gondar as a tourist destination has inevitably led to a surge in demand for food services provided by hotels and restaurants. While the burgeoning hospitality industry contributes to the economic vitality of the region, it concurrently raises concerns regarding the potential health risks associated with foodborne illnesses. As the demand for food services escalates, so does the need to scrutinize the factors influencing food safety, particularly in the context of intestinal parasites. Individuals who are directly engaged in food preparation and service play a crucial role in upholding food hygiene. The close interaction between food handlers and the food they prepare makes this group a critical focus for understanding and mitigating the risk of foodborne parasitic infections.

The distinct circumstances of Gondar City underscore the urgency of appraising the prevalence of intestinal parasites among food handlers. The potential impact of parasitic infections on both the local population and the influx of tourists underscores the urgency of this research. By exploring intestinal parasites among food handlers, the research aims to provide empirical data that can inform evidence-based interventions. The findings of this study will help develop targeted measures to enhance food safety practices, thereby safeguarding public health in Gondar City.

Furthermore, the insights gained from this research can contribute to the broader discourse on food safety in Ethiopia, offering valuable lessons and recommendations that may apply to other regions facing similar challenges. The identification of specific risk factors and the development of interventions tailored to the local context can serve as a model for enhancing food safety practices in other cultural and historical centers across the country.

## Materials and methods

### Study area, design, and period

**Figure 1:**
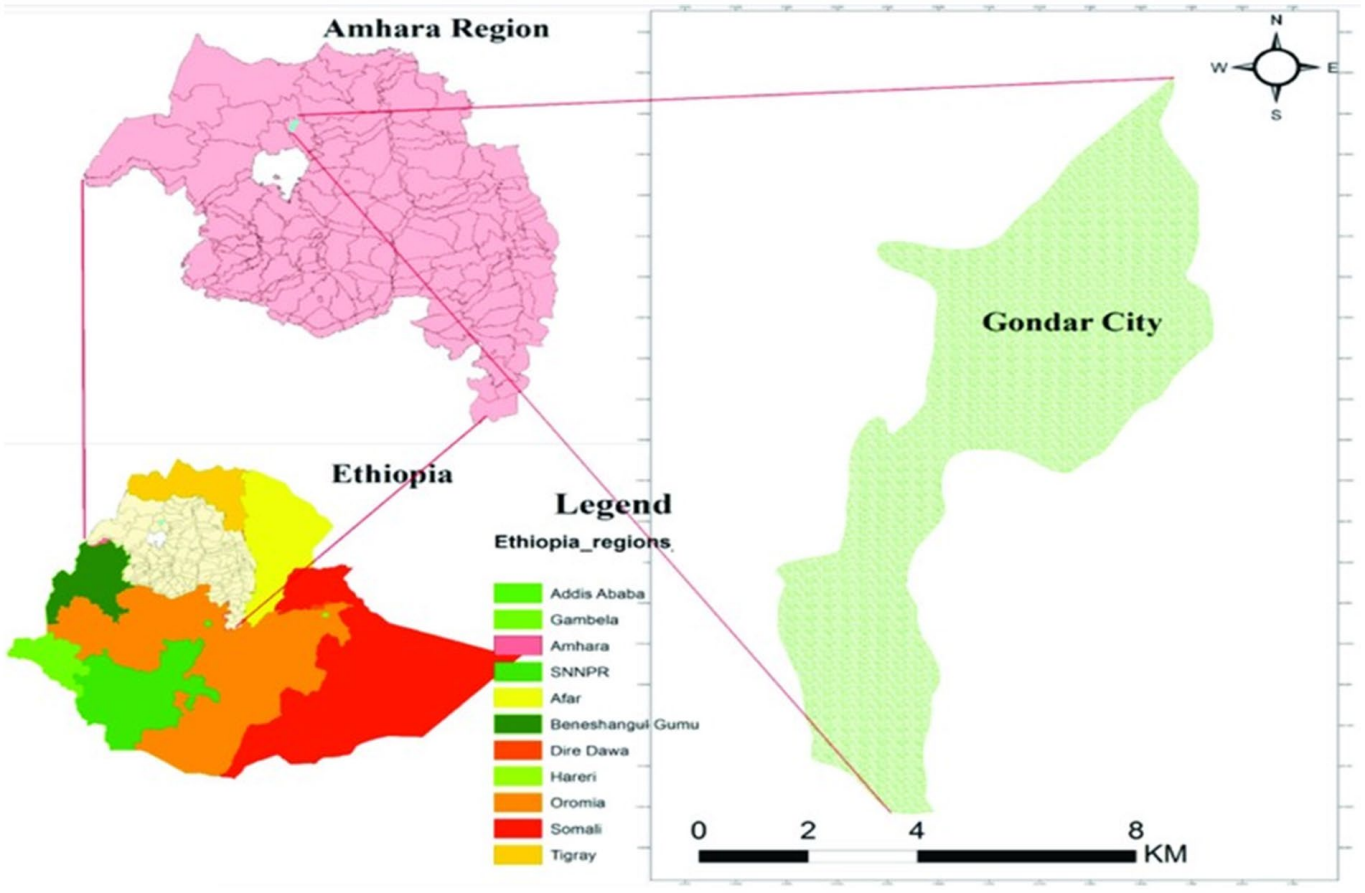
Illustrates a visual representation of the study area, Gondar City. Source: Tamiru et al. (38).

A cross-sectional study was conducted from February to April 2020 in Gondar City. The city is found in the Amhara regional state, approximately 747 kilometers to the northwest of Addis Ababa (the capital city of Ethiopia) and 182 kilometers from Bahir Dar. According to the Central Statistical Agency’s 2017 population projection, the city’s total population hovers around 360,600, with roughly 176,593 being males (36, 37). The city is administratively divided into 24 ‘kebeles,’ the smallest administrative units. Furthermore, Gondar is divided into several administrative divisions, each playing a significant role in public health and sanitary issues. These divisions include the Gondar City Administration Health Department, the Gondar City Administration, and individual district offices. The Gondar City Administration Health Department is primarily responsible for overseeing health-related initiatives within the city, including the management of health risks associated with food handling. They conduct regular inspections, provide training for food handlers, and implement regulatory measures to ensure food safety. The Gondar City Administration is responsible for the broader governance of the city, including the provision of public services and infrastructure that indirectly relate to public health. They work in close collaboration with the Health Department to address health challenges. Each district office within Gondar City has its administrative roles in managing health-related activities in their respective districts. They are responsible for implementing health initiatives at a district level, collecting data, and reporting to the zonal Health Department office. The exact number of food handlers in each food establishment could not be determined due to high turnover (see Figure 1).

### Populations

The source population consisted of all food handlers who worked at hotels and restaurants in Gondar City during the study period. The study population included only those food handlers who met the inclusion criteria, which were: willingness to participate in the study and no intake of anti-parasite drug (s) in the 2 weeks before the start of the study.

### Inclusion and exclusion criteria

The inclusion criteria encompass all individuals directly involved in handling, preparing, or serving food within Gondar City Hotels and Restaurants and willing to take part in the study. The exclusion criteria were study participants who took antiparasitic medication within 2 weeks before the data collection period, and those individuals with chronic illnesses affecting the gastrointestinal tract were excluded.

### Sample size determination and sampling technique

The minimum sample size for the study was calculated using a single population proportion formula based on the assumption of a 5% expected margin of error (d = 0.05), 95% confidence interval (z = 1.96), and 8.7% prevalence from a previous study conducted in Wolaita Sodo town (39). It is important to note that Wolaita Sodo town, like Gondar city, is a zonal town. We expect the populations of the two towns to be similar, which forms the basis for our sample size estimation.


$$\frac{n_{i}={\left(Z\alpha /2\right)}^{2}pq}{d^{2}}$$

$$\frac{n_{i}={\left(1.96\right)}^{2}\;0.087\;\left(1-0.087\right)}{{\left(0.05\right)}^{2}}$$

$$n_{i}\approx 125^{*}\;2.$$

$$n_{i}\approx 250.$$

Since the total number of the source population was less than 10,000, the correction formula was applied to adjust the final sample size (nf):


nf=1+niniN=1+2502508417≈244

Finally, considering a 10% non-response rate, the final sample size was estimated to be 269 study participants. The participants were selected from five sub-cities using multistage sampling. A complete list of catering establishments was obtained from the Gondar Trade Administration office. The establishments in the selected sub-cities were stratified into hotels and restaurants. The required sample size was then proportionally allocated to each stratum, and a random sampling technique was employed to include the necessary number of participants from each category.

### Data data collection

Data about socio-demographic characteristics and other variables were collected using a structured pre-tested questionnaire. The questionnaire contained information on age, gender, marital status, year of service, educational level, previous training, and practice of hand washing of the food handlers.

### Stool sample collection and transportation

Stool samples were collected from food handlers using standard procedures. The collection took place in the morning because this time of the day enhances the detection of parasites in the stool. Each participant was provided with a clean, dry, leak-proof, and labeled plastic stool cup for self-collection. The study participants were instructed to utilize the provided applicator stick to pick up a piece of stool, place it in the clean plastic container provided, and deliver it immediately. A minimum of 10 grams of stool specimens were collected from each study participant. This quantity is necessary to ensure that an adequate amount of the sample is available for diagnostic testing. The containers were tightly closed to prevent leakage and contamination. Then the stool samples were immediately stored in a cold box after labelling them with a code on the outer surface of the plastic cup. The collected samples were then transported at room temperature to the medical parasitology laboratory of the University of Gondar Comprehensive Specialized Hospital within an hour of collection. This rapid transportation minimizes the risk of sample degradation and ensures that the samples reach the laboratory in optimal condition for analysis.

### Direct wet mount preparation

A drop of physiological saline was applied to one slide, while the other received Dobell’s iodine. Using an applicator stick, a small (equivalent to the size of a matchstick head) quantity of stool specimen was evenly spread over the separate glass slides with physiological saline and iodine. The specimens were then covered with a cover glass and scrutinized for the presence of helminth eggs, larvae, ciliates, cysts, and oocysts under 40× objectives (40).

### Formol ether concentration techniques

An estimated 1 g of formed stool sample or 2 mL of watery stool was emulsified in about 4 mL of 10% formol water contained in a screw-cap bottle. Furthermore, 3 mL of 10% formol water was added and mixed well by shaking. The emulsified feces were sieved, and the suspension was transferred to a conical centrifuge tube. About 3 mL of diethyl ether was added, and the tube was mixed for 1 min. Then, the samples were centrifuged at 3000 rpm for 1 min. The tubes were inverted to discard the ether, fecal debris, and formol water, leaving behind the sediment. The bottom of the tubes was taped to re-suspend and mix the sediment. The sediment was transferred to a slide covered with a cover glass and examined microscopically using 40× objective lenses (40).

### Data management and analysis

After ensuring completeness, data were entered using EpiData version 3.1 and exported to SPSS 20 for analysis. Descriptive statistics, such as frequencies, percentages, and mean, were used primarily to summarize the findings. Further, bivariate and multivariate logistic regression analyses were performed to investigate the relationships between the predictors and outcome variables. All variables with a *p*-value of ≤0.2 in the bivariate analysis were transferred into the multivariable logistic regression model to adjust for possible confounding variables. Finally, variables with a p-value of <0.05 in the final analysis were considered to indicate significant associations.

### Ethical consideration

Ethical clearance was granted by the University of Gondar, School of Biomedical and Laboratory Sciences ethics review committee (SBMLS/870/10), and support letters were written from the North Gondar Hotel and Tourism Management Bureau before data collection. Further permission was obtained from the Zonal Health Department office. A formal letter was also written from the Municipality of the city to food establishments and written informed consent was obtained from each study participant before commencing the data collection. To ensure the privacy of the study participants during interviews, all data collection processes were conducted in an isolated area. Furthermore, participants were informed that all data and samples obtained from them would be kept confidential. Participants who tested positive for intestinal parasitic infection were referred to a nearby health facility for appropriate treatment of the disease.

## Results

### Socio-demographic data

In this study, a total of 257 food handlers participated in the study with a 96% response rate. From this 91.8% (238) of them were females. The median age of the study participants was 25.71 ± 5.37 years and the majority 74.7% (192) were in the age group of 20–40 years. Nearly one-fourth (63) of the study participants were illiterate, while 47.4% (171) had secondary school education or above.

### Hygienic practice of the study participants

The majority (89.1%) of the study participants had worked for less than 5 years. Concerning training, only 23% (59) of them had training certificates on food handling techniques. Slightly higher than three-fourths (200) of the study participants had hand washing practice using water only. Slightly higher than one-third (100) were regularly supervised. The majority 64.6% (166) of the study participants had hair covering practice. Regarding fingernail status, the majority 65.8% (169) of the study participants were observed not clean/ not cut (Table 1).

**Table 1:** Hygienic practice of study participants in Gondar City, Ethiopia, 2020.

Variables	Variable category	Number (*N*)	%
Training on food handling practice	Yes	59	23.0
	No	198	77.0
Year of service	< 1 year	110	42.8
	1–5 years	119	46.3
	6–10 years	23	8.9
	>11 years	5	1.9
Supervision	Regularly supervised	100	38.9
	Intermittently supervised	118	45.9
	Never supervised	39	15.2
Hair covering practices	Yes	91	35.4
	No	166	64.6
Wearing jewelry	No	177	68.9
	Yes	80	31.1
Hand washing practice	With soap & water	57	22.2
	With water only	200	77.8
Wearing gown	Yes	133	51.8
	No	124	48.2
Fingernail status	Yes, clean and cut	88	34.2
	No, not clean not cut yet	169	65.8
Status of fly infestation during data collection	Not seen	180	70.0
	Seen	77	30.0

### Prevalence of intestinal parasites

**Figure 2:**
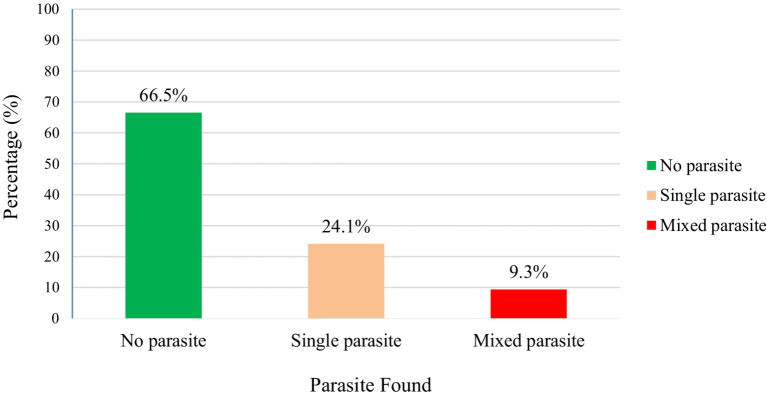
Intestinal parasites found from food handlers at Gondar City, Northwest Ethiopia, 2020.

The overall prevalence of IPIs among food handlers in Gondar City was 33.5% (86/257) with a 95% confidence interval (CI) of 28.0–39.5%. Of these, 62 (24.1%) were infected by a single parasite, while 24 (9.3%) had mixed infections (Figure 2).

Nine types of intestinal parasites were found in food handlers’ stools. Among these, *E. histolytica/dispar* was the predominant 13.7% (35/257) parasite, followed by *A. lumbricoides* 11% (28/255), hookworms 6.7% (17/255), and *S. mansoni* and *G. lamblia* each accounting for 4.3% (11/257) (Table 2).

**Table 2:** Types and prevalence of intestinal parasites observed in stool specimens of food handlers in Gondar City, 2020 (*N* = 257).

Type of parasite identified	Number	Prevalence (%)
*E. histolytica*/*dispar*	21	8.2
*A. lumbricoides*	17	6.6
Hookworm species	9	3.5
*S. mansoni*	6	2.3
*E. histolytica*/*dispar* and *A. lumbricoides*	5	1.9
*G. lamblia*	4	1.6
*Taenia species*	4	1.6
*E. histolytica*/*dispar* and *G. lamblia*	3	1.2
*E. histolytica*/*dispar* and *Hookworm species*	3	1.2
*A. lumbricoides* and *S. mansoni*	3	1.2
*Hookworm species* and *G. lamblia*	2	0.8
*S. mansoni* and *E. histolytica/dispar*	2	0.8
*G. lamblia* and *A. lumbricoides*	2	0.8
*Hookworm* and *T. trichuira*	2	0.8
*E. vermicularis*	1	0.4
*S. stercoralis* and *E. histolytica/dispar*	1	0.4
*A. lumbricoides* and *Hookworm*	1	0.4

### Factors associated with intestinal parasitic infections

The bivariate analysis revealed that fingernail status and supervision were marginally associated with intestinal parasitic infections (*p* < 0.2), while educational status and training on food handling were significantly associated with them. The multivariable analysis showed that, after adjusting for potential confounders, illiteracy and having a secondary school education were the key factors contributing to IPIs. Compared to food handlers with college education or above, illiterate food handlers had 7.37 times higher odds of being infected (95% CI: 1.54–35.16), and food handlers with secondary school education had 5.97 times higher odds of being infected (95% CI: 1.31–27.12) (*p* < 0.05) (Table 3).

**Table 3:** Association between intestinal parasite infections and potential factors among food handlers, 2020.

Variables	Variable category	Intestinal parasite		Prevalence of intestinal parasite	
		Yes	No	COR (95% CI)	AOR (95% CI)
Gender	Male	8	13	0.80 (0.32–2.02)	
	Female	78	158	1	–
Age in years	<20	21	36	0.97 (0.21–4.49)	
	20–40	62	130	0.80 (0.18–3.43)	
	>40	3	5	1	
Educational status	Illiterate	28	35	8.40 (1.81–38.92)	7.37 (1.54–35.16)^*^
	Primary	20	52	4.04 (0.87–18.83)	3.55 (0.74–17.08)
	Secondary	36	63	6.00 (1.33–27.08)	5.97 (1.31–27.12)^*^
	Collage and above	2	21	1	1
Work experience	<1 year	31	79	0.59 (0.09–3.69)	–
	1–5 years	46	73	0.95 (0.15–5.87)	
	6–10 years	7	16	0.66 (0.09–4.84)	
	>10 years	2	3	1	
Training on food handling	Yes	10	40	1	1
	No	76	129	1.09 (0.59–2.03)	0.61 (0.27–1.38)
Supervision	Regularly	31	69	1	1
	Intermittently	38	80	1.06 (0.60–1.88)	0.89 (0.47–1.66)
	Never	17	22	1.72 (0.80–3.68)	1.44 (0.62–3.33)
Hand washing practice	Using water and soap	67	133	1	
	Using only water	19	38	0.99 (0.53–1.85)	–
Wearing gown	Yes	46	87	1	
	No	40	84	0.90 (0.54–1.51)	
Hair cover	Yes	32	59	1	
	No	54	112	0.89 (0.52–1.52)	–
Jewelry	Yes	26	54	1	
	No	60	117	0.94 (0.54–1.65)	
Fingernail status	Not trimmed	62	107	1.55 (0.88–2.72)	0.70 (0.38–1.30)
	Trimmed	24	64	1	1
Flies	Seen	26	51	0.98 (0.0.56–1.73)	–
	Not seen	60	120	1	

^*^Statistically significant.

## Discussion

Intestinal parasitic infections pose a significant public health challenge, especially in developing countries. Food handlers, particularly those working in hotels and restaurants, play a crucial role in the transmission of these infections. This study aimed to estimate the prevalence of intestinal parasites among food handlers and identify the associated factors.

The overall prevalence of IPIs among food handlers in this study was found to be 33.5%. This finding aligns with systematic review and meta-analysis studies from Ethiopia conducted at different times, which reported prevalence rates of 29.2 and 33.6% (13, 41). Other studies from southern Ethiopia (14, 29, 42), Jimma town (43), Sudan (44), and Kenya (45) reported similar prevalence rates ranging from 29.4 to 39.2%. These results underscore the potential for food handlers to transmit intestinal parasites to consumers, emphasizing the importance of implementing screening and mass drug administration programs to curb the spread of infections within food establishments. However, lower prevalence rates were observed in other studies, ranging from 14.5 to 25.3% in different parts of Ethiopia, Saudi Arabia, Iran, and Sudan (11, 31–33, 46–53). The disparity in prevalence rates across these locations could be influenced by several factors such as the level of awareness regarding intestinal parasites and their transmission, the educational background of food handlers, and the stringency and effectiveness of regulatory enforcement in the food industry could greatly impact the prevalence of such infections. Moreover, the epidemiology of the disease itself could lead to variations in prevalence. The presence of different species of parasites, their life cycles, and the local environmental conditions all contribute to this epidemiological complexity (54). Additionally, the methodologies and accuracy of laboratory techniques employed, along with the expertise of the laboratory personnel, are critical factors that determine the reliability of the prevalence figures reported. These multifaceted factors underscore the complexity of controlling IPIs among food handlers.

Significantly higher prevalence rates were observed in various locations, notably in Turkey (59%), Gondar (45.7%), Mettu town (44.6%), south Ethiopia (41%), Addis Ababa University student’s cafeteria (45.3%), Pakistan (59.8%), and Nekemte town (52.1%) (17, 21, 55–59). The disparities in prevalence might be attributed to various factors, including differences in laboratory techniques, regional epidemiology, weather conditions, levels of awareness and education among study participants, and variations in regulatory enforcement.

The prevalence of mixed infections of intestinal parasitic infections among food handlers is alarmingly high at 9.3%. This percentage is notably greater than what has been observed in various regions of Ethiopia, such as Tigray, the University of Southern Ethiopia, Southern Ethiopia, Wolaita Sodo, and Addis Ababa, which reported prevalence rates of 3.4, 3.3, 0.6, 0.8, and 3.4%, respectively, (14, 17, 29, 47). This prevalence of mixed infections in the current study is more than double the highest rate seen in these other areas. This data suggests that the problem is not evenly distributed, and that certain populations or areas might be more vulnerable to these parasitic infections than others. There might be various factors contributing to this discrepancy, such as differences in hygiene practices, access to clean water, exposure to contaminated food, or local health policies. The high prevalence rate of mixed infections from the current study is concerning due to the complexity it brings to treatment. This complicates treatment as different parasites may require different therapeutic approaches. Furthermore, the presence of multiple parasites can exacerbate the severity of the disease and lead to more severe health outcomes. This situation emphasizes the urgent need for intervention.

The current study found that *E. histolytica/dispar* and *A. lumbricoides* were the most identified parasites, with prevalence rates of 8.2 and 6.6%, respectively. These were followed by Hookworm species (3.5%) and *S. mansoni* (2.3%). The prevalence of these parasites is not uniform and shows considerable variation across different geographical locations and study populations. For instance, in Yebu, the prevalence of *A. lumbricoides* was reported to be 17.8%, which is significantly higher than our findings (6). Similarly, in Jimma, both *A. lumbricoides* and *E. histolytica/dispar* were found at 16.0 and 4.3%, respectively (60). In Arba Minch, the prevalence rates for *E. histolytica/dispar* and *A. lumbricoides* were 14 and 9.27%, respectively, which are also higher than our current study results (14). A study from Pakistan reported even higher prevalence rates for *A. lumbricoides* and *E. histolytica/dispar* at 55.8 and 14.2%, respectively (61). Moreover, a staggering 70.8% prevalence of *E. histolytica/dispar* was reported from Addis Ababa (62).

Several factors such as local sanitation practices, the availability of clean water, public health initiatives, and the level of community awareness about parasitic infections can greatly influence the prevalence rates. For example, areas with poor sanitation and hygiene practices are more likely to have higher rates of parasitic infections due to the increased risk of fecal-oral transmission. Additionally, the climate and environmental conditions of a region can affect the lifecycle of parasites and their transmission dynamics, leading to regional differences in prevalence. Furthermore, the methodology of the studies, including the diagnostic techniques used, can also contribute to the variability in reported prevalence rates. Some diagnostic methods may have higher sensitivity and specificity, leading to more accurate detection of parasitic infections.

The study revealed a significant correlation between the educational background of food handlers and the prevalence of intestinal parasites. It was found that food handlers who were illiterate had a risk of 7.37 times greater than that of individuals with a college or higher level of education. Similarly, those with a secondary school education were found to have a 5.97 times higher chance of getting an infection when compared to their counterparts with a college education or above. Interestingly, food handlers with primary education exhibited a lower risk of infection than both their uneducated peers and those with secondary education. This unexpected finding warrants further investigation into the specific factors influencing these relationships. It was evident that, despite having a high level of education, some food handlers were found to be infected with intestinal parasites (63). Despite the complexities, the study highlights the critical role that education plays in fostering proper hygiene practices among food handlers. It underscores the need for educational programs specifically tailored to this group to diminish the prevalence of parasitic infections. Such interventions are crucial, as they not only improve the health standards of the food handlers themselves but also safeguard the well-being of the wider community they serve.

### Treatment strategies and future perspectives

Early diagnosis and prompt treatment are key, with screening programs essential for early detection and reducing transmission. Furthermore, due to the increasing drug resistance and limited options for controlling parasitic infections, it is vital to investigate drug efficacy and develop alternative preventive measures (2). Effective management of mixed infections requires customized treatments and in-depth knowledge of parasites by healthcare providers. To tackle IPIs, understanding local factors like awareness and regulations is vital for creating targeted interventions. Regular monitoring of food handlers is important for controlling infection spread and informing policy. Educational programs for food handlers, focusing on hygiene and prevention, are crucial in reducing infections and improving public health.

### Strengths

The study demonstrated strength in its methodology by utilizing both direct wet mount preparations and formol-ether concentration techniques for the microscopic examination of stool samples. The incorporation of the concentration technique notably increased the sensitivity of parasite detection, thereby enhancing the precision and reliability of the study’s findings. Additionally, the study exhibited a proactive approach to participant welfare by ensuring that individuals who tested positive for intestinal parasitic infections were promptly linked to nearby health facilities for necessary treatment.

### Limitations

The study presented several limitations that impacted its findings. Firstly, the cross-sectional design of the study was a significant limitation as it restricted the ability to establish causal relationships between various factors and the outcomes related to intestinal parasitic infections. Secondly, the reliance on self-reported data from the food handlers could have introduced biases such as recall bias and social desirability bias. This is evident in instances where food handlers might have overestimated their frequency of hand-washing or underreported symptoms associated with intestinal parasitic infections. Additionally, the study’s methodology of using a single stool sample from each participant may not have accurately represented the true prevalence of intestinal parasitic infections. The intermittent or low shedding rate of some parasites could lead to false negative results, suggesting that repeated stool examinations or more sensitive diagnostic methods might be necessary to verify the food handlers’ infection status accurately. Lastly, the study did not evaluate the quality and availability of water and sanitation facilities within the food establishments, which are critical factors that can influence the risk of intestinal parasitic infections. Furthermore, there was no assessment of the food handlers’ knowledge, attitude, and practice regarding food safety and hygiene, which are essential components that can affect their behavior and, consequently, their infection status.

## Conclusion

The study uncovered a high prevalence of intestinal parasitic infections among food handlers with a notable prevalence of mixed infections. Among the isolated infections, *E. histolytica/dispar* and *A. lumbricoides* were identified as the most commonly prevalent parasitic infections among study participants. The study also highlighted a significant correlation between educational status and the risk of infection, emphasizing the potential public health threat posed to consumers and the community.

## Recommendations

- The Federal Ministry of Health and other stakeholders should implement policies and programs that aim to improve the awareness of food handlers.
- The Federal Ministry of Education should implement policies and programs that aim to improve the educational status of food handlers.
- The Gondar city administration office should design and periodically provide training to food handlers on the modes of transmission, prevention, and treatment of parasitic infections, as well as the importance of personal hygiene and sanitation.
- Zonal health department and city administration offices should conduct periodic screening and treatment of food handlers and enforce strict compliance with regulations and standards.
- Researchers should conduct large-scale studies to explore factors influencing the compliance and adherence of food handlers to preventive measures, along with assessing the cost-effectiveness and feasibility of screening and mass drug administration programs.
- Zonal health departments and city administration offices should conduct periodic inspections to ensure compliance with food safety standards and regulations.

## Data availability statement

The original contributions presented in the study are included in the article/supplementary material, further inquiries can be directed to the corresponding author.

## Ethics statement

The studies involving humans were approved by University of Gondar, School of Biomedical and Laboratory Sciences Research and Ethics Review comitee. The studies were conducted in accordance with the local legislation and institutional requirements. The participants provided their written informed consent to participate in this study.

## Author contributions

MG: Conceptualization, Data curation, Formal analysis, Funding acquisition, Resources, Visualization, Writing – original draft. GB: Conceptualization, Data curation, Writing – original draft. AA: Investigation, Supervision, Writing – review & editing. WA: Conceptualization, Data curation, Supervision, Writing – review & editing. TD: Data curation, Formal analysis, Writing – original draft, Writing – review & editing.
